# Under the name of “Lua”: revisiting genetic heterogeneity and population ancestry of Austroasiatic speakers in northern Thailand through genomic analysis

**DOI:** 10.1186/s12864-024-10865-3

**Published:** 2024-10-14

**Authors:** Jatupol Kampuansai, Tanapon Seetaraso, Maneesawan Dansawan, Suwapat Sathupak, Wibhu Kutanan, Metawee Srikummool, Angkana Inta

**Affiliations:** 1 Department of Biology, Faculty of Science, Chiang Mai University, Chiang Mai, Thailand; 2 Department of Biology, Faculty of Science, Naresuan University, Phitsanulok, Thailand; 3 Center of Excellence for Innovation and Technology for Detection and Advanced Materials (ITDAM), Naresuan University, Phitsanulok, Thailand; 4 Department of Biochemistry, Faculty of Medical Science, Naresuan University, Phitsanulok, Thailand; 5 Center of Excellence in Medical Biotechnology, Faculty of Medical Science, Naresuan University, Phitsanulok, Thailand

**Keywords:** Lua, Palaungic, Austroasiatic, Ancestry, Heterogeneity, Ethnic group, Northern Thailand

## Abstract

**Background:**

Austroasiatic (AA)-speaking populations in northern Thailand are of significant interest due to their status as indigenous descendants and their location at the crossroads of AA prehistoric distribution across Southern China, the Indian Subcontinent, and Mainland Southeast Asia. However, the complexity of ethnic identification can result in inaccuracies regarding the origin and migration history of these populations. To address this, we have conducted a genome-wide SNP analysis of 89 individuals from two Lavue and three Lwa-endonym populations. We then combined our outcomes with previously published data to elucidate the genetic diversity and clustering of AA groups in northern Thailand.

**Results:**

Our findings align with existing linguistic classifications, revealing different genetic compositions among the three branches of the Mon-Khmer subfamily within the AA family: Monic, Khmuic, and Palaungic. Although the term “Lua” ethnicity is confusingly used to identify ethnic groups belonging to both Khmuic and Palaungic branches, our genomic data indicate that the Khmuic-speaking Lua living on the eastern side of the region are relatively distant from the Palaungic-speaking Lavue and Lwa populations living on the western side. The Lavue populations, primarily inhabiting mountainous areas, exhibit a genetic makeup unique to the AA family, with a close genetic relationship to the Karenic subgroup of the Sino-Tibetan language family. Conversely, the Lwa and Blang populations, residing in lowland river valleys, display genetic signatures resulting from admixture with Tai-Kadai-speaking ethnic groups.

**Conclusion:**

Utilizing genome-wide SNP markers, our findings indicate genetic heterogeneity among the Lua, Lavue, and Lwa ethnic groups. The intricate interplay of genetics, cultural heritage, and historical influences has shaped these ethnic communities. Our study underscores the importance of accurate ethnic classifications, emphasizing the use of self-identified endonyms, names created and used by the ethnic groups themselves. This approach respects the AA communities in northern Thailand and acknowledges their significant contributions to advancing our understanding of genetic anthropology.

**Supplementary Information:**

The online version contains supplementary material available at 10.1186/s12864-024-10865-3.

## Background

The Austroasiatic (AA)-speaking people are an ethnolinguistic group of people living in Southeast Asia and parts of South Asia. They possess a diverse history spanning millennia, which encompasses various cultures, languages, and societies. The AA language family stands out as a primary language group in Southeast Asia, alongside Tai-Kadai (TK), Sino-Tibetan (ST), Austronesian (AN), and Hmong-Mien (HM) languages. The ancestors of AA-speaking populations were likely Neolithic rice farmers residing in southern China around 7,000 years ago [1]. This population embarked on multiple migration waves into Southeast Asia, a process that would have begun as early as the Neolithic era [2, 3]. This migration was likely driven by a combination of factors, including population growth, environmental shifts, and the expansion of agricultural practices [4]. The development of settled agricultural societies, facilitated by the spread of agriculture, played a pivotal role in the emergence of complex civilizations in Southeast Asia. One notable example is the Dvaravati Mon civilizations that flourished around the 7th century AD in Thailand’s Chao Phraya Valley, a region renowned for its rice production [5].

The northern part of Thailand holds strategic importance as it serves as a vital gateway connecting Thailand to neighboring countries such as Myanmar, Laos, and China. This area has served as a historical crossroads for the southward migration of AA people from East to Southeast Asia since ancient times. In northern Thailand, AA-speaking populations are recognized as indigenous groups that have descended from prehistoric populations [6]. All of them are linguistically classified within the Mon-Khmer subfamily of the AA family, and have further been divided into three branches: Monic, Khmuic, and Palaungic [7]. The Monic branch comprises only the Mon ethnicity, while the majority of present-day AA speakers in northern Thailand belong to the Palaungic and Khmuic branches, categorized under the Northern Mon-Khmer subfamily with estimated populations of 20,483 and 58,405 people, respectively [8].

**Fig. 1:**
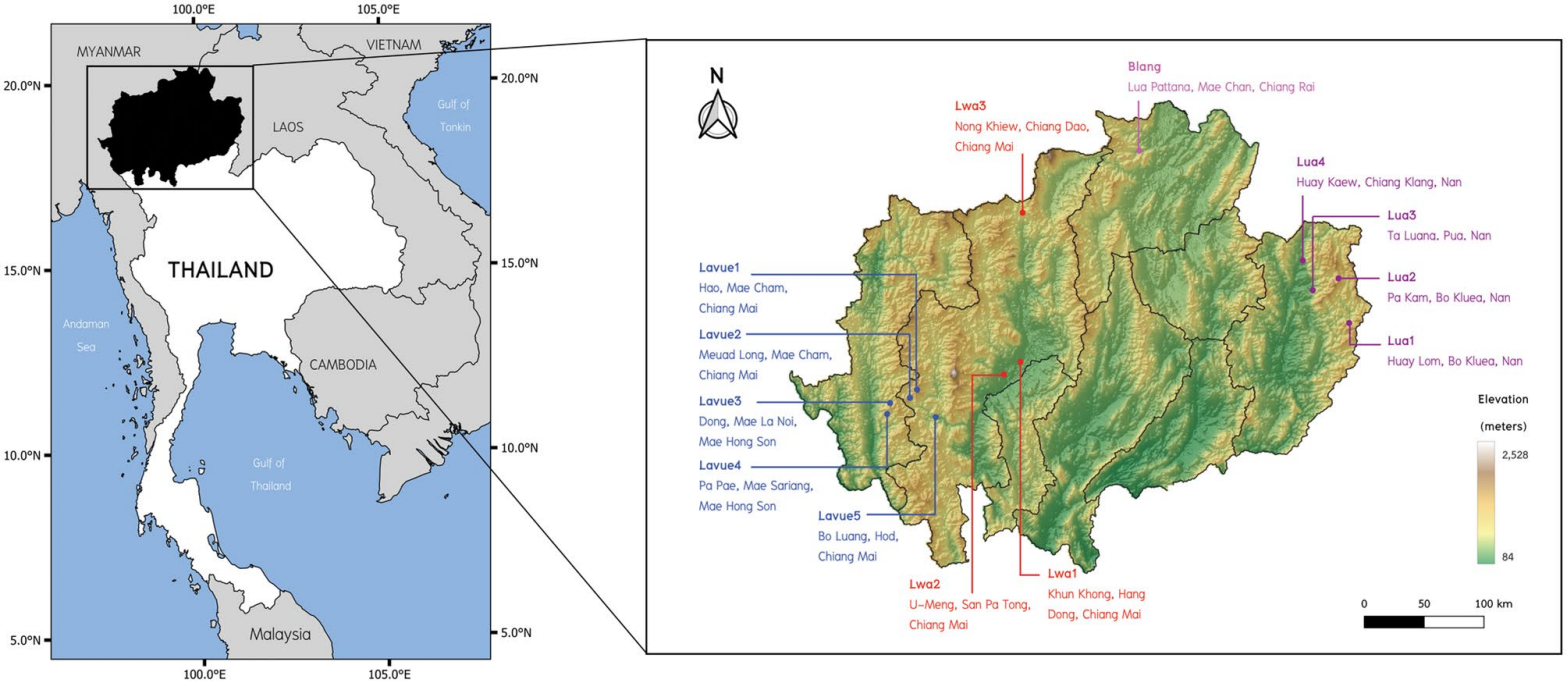
Geographical distribution of AA samples commonly referred to as the Lua collective name. The colored text on the map of northern Thailand represents the Lua or related ethnonym. Background map was created using QGIS 3.6.0 (http://www.qgis.org/)

Among the identification of AA-speaking ethnicities residing in northern Thailand, the term “Lua” is the most confusing in various research works because it has been used to refer to certain ethnic groups in the Khmuic branch living in the eastern part of the northern region near the border with Laos. Simultaneously, some researchers use it to describe the Palaungic-speaking community in the highland areas of Chiang Mai and Mae Hong Son Provinces, located in the western part of northern Thailand near the border with Myanmar (Fig. 1; Table 1). In fact, each of these ethnicities has their own endonym (a name created and used by the ethnic group itself) and differs significantly in terms of ancestry and historical background. The confusion surrounding the term “Lua” arises from its similarity to other closely related ethnonyms, such as Lua, Lawa, Lavue, and Lavua [9]. Since they all speak languages belonging to the AA family, outsiders often overlook these differences and assume they are the same ethnic group. For example, “Lua” or “Lawa” is an exonym (a name for an ethnic group created by another group of people) commonly used by Northern Thai, Central Thai, and Christians to refer to Palaungic-speaking people, but it is not an endonym used by these people to identify themselves. Furthermore, in some historical contexts, the term “Lua” does not specifically denote any ethnic group but rather serves as a derogatory term used by rulers to distinguish indigenous inhabitants residing outside urban areas [10].

**Table 1:** Information pertaining to the AA-speaking population residing in northern Thailand, for whom genome-wide autosomal data is accessible

Code	Ethnic name		Location (village, district, province)	Language branch	No. of samples	Reference
	endonyms	exonyms				
Lwa1	Lwa*, Lua	Lua, Lawa	Khun Khong, Hang Dong, Chiang Mai	Palaungic	20	This study
Lwa2	Lwa*, Lua	Lua, Lawa	U-Meng, San Pa Tong, Chiang Mai	Palaungic	15	This study
Lwa3	Lwa*, Lua	Lua, Lawa, Pareak	Nong Khiew, Chiang Dao, Chiang Mai	Palaungic	14	This study
Lavue1	Lavue, Laveue	Lua, Lawa	Hao, Mae Cham, Chiang Mai	Palaungic	25	This study
Lavue2	Lavue, Laveue	Lua, Lawa	Meuad Long, Mae Cham, Chiang Mai	Palaungic	15	This study
Lavue3	Lavue, Laveue	Lua, Lawa (Western)	Dong, Mae La Noi, Mae Hong Son	Palaungic	10	Changmai et al., 2022 [20]
Lavue4	Lavue, Laveue	Lua, Lawa (Western)	Pa Pae, Mae Sariang, Mae Hong Son	Palaungic	9	Kutanan et al., 2021 [19]
Lavue5	Lavue, Laveue	Lua, Lawa (Eastern)	Bo Luang, Hod, Chiang Mai	Palaungic	10	Kutanan et al., 2021 [19]
Blang	Blang	Lua, Blang, Tai Doi, Sam Tao	Lua Pattana, Mae Chan, Chiang Rai	Palaungic	9	Kutanan et al., 2021 [19]
Dara-ang	Dara-ang	Palaung	Nor Lae, Chiang Dao, Chiang Mai	Palaungic	9	Kutanan et al., 2021 [19]
Lua1	Lua	Lua, Htin	Huay Lom, Bo Kluea, Nan	Khmuic	13	Kampuansai et al., 2023 [12]
Lua2	Lua	Lua, Htin	Pa Kam, Bo Kluea, Nan	Khmuic	14	Kampuansai et al., 2023 [12]
Lua3	Lua	Lua, Htin, Mal	Ta Luang, Pua, Nan	Khmuic	10	Lipson et al., 2018 [2]
Lua4	Lua	Lua, Htin, Pray	Huay Kaew, Chiang Klang, Nan	Khmuic	11	Kutanan et al., 2021 [19]
Khamu1	Kmhmu, Khamu	Khamu, Khammu	Pang Sa, Tha Wang Pha, Nan	Khmuic	17	Kampuansai et al., 2023 [12]
Khamu2	Kmhmu, Khamu	Khamu, Khammu	Chon Dan, Song Khwae, Nan	Khmuic	16	Kampuansai et al., 2023 [12]
Khamu3	Kmhmu, Khamu	Khamu, Khammu	Huay Puk, Mueng, Nan	Khmuic	21	Kampuansai et al., 2023 [12]
Khamu4	Kmhmu, Khamu	Khamu, Khammu	Huay Sataeng, Thung Chang, Nan	Khmuic	10	Kutanan et al., 2021 [19]
Mlabri	Mlabri	Mlabri	Huay Yuak, Wiang Sa, Nan	Khmuic	9	Lipson et al., 2018 [2]
Mon_NT	Mon	Mon	Nong Du, Pa Sang, Lamphun	Monic	9	Changmai et al., 2022 [20]

*The present-day villagers pronounce their endonym as “Lua”. However, to distinguish them from the “Lua” ethnicity of Nan Province, we chose to use “Lwa”, which is indicative of their historic ethnonym

This misidentification of ethnicities has implications for genetic anthropological studies, as some research studies may incorrectly consider these distinct groups to be of the same ethnicity and analyze data without recognizing their differences. This issue is particularly significant because the AA people, who are the original inhabitants of Mainland Southeast Asia, represent a population that could be highly relevant to the extensive ongoing exploration of ancient DNA studies. While some previous studies have attempted to clarify the genetic classification of AA speakers in northern Thailand [11, 12], the limited number of samples in the Palaungic branch, which is the most diverse and complex within this language family, has hindered the revelation of their genetic ancestry and history. Therefore, we have generated new genome-wide data for AA-speaking populations in northern Thailand, specifically targeting all ethnic endonyms within the Palaungic branch, thereby aiming to elucidate the intricacies of a subgroup classification within the AA language family.

## Methods

### Sample collection and quality control

We collected samples from 95 unrelated individuals living in five villages in Chiang Mai and Mae Hong Son Provinces of northern Thailand. The participants were healthy subjects over the age of 20, of Palaungic-speaking ethnicity, and had no known ancestors from other recognized ethnic groups for at least three generations. We collected personal data through form-based oral interviews for endonyms, self-reported unrelated lineages, languages, and migration histories. Buccal specimens were collected, and genomic DNA was extracted using the Gentra Puregene Buccal Cell Kit (Qiagen, Germany) following the manufacturer’s instructions. Detailed sample information is listed in Table 1, and the geographic locations of sampling are shown in Fig. 1.

Accordingly, to reduce confusion regarding terminology and classification under the “Lua” ethnicity, we will primarily use the self-identifying endonyms that residents use to refer to themselves and will further refer to names used in previous reports.

## Genotyping and data preparation

Genotyping was carried out using the Affymetrix Axiom Genome-Wide Human Origins array [13] at AtlasBiolab in Germany. A total of 93 samples were genotyped for 629,721 genetic markers on the hg19 version of the human reference genome, with a genotype call rate of at least 97%. We used PLINK version 1.90b5.2 [14] to remove genetic markers and individuals with more than 5% missing data, as well as to exclude mitochondrial DNA and sex chromosomes from our analysis. Loci that did not pass the Hardy-Weinberg equilibrium test (p-value < 0.00005), or those that had more than 5% missing data within any population, were also excluded. Additionally, we used KING 2.3 [15] to assess individual relationships and removed one person from each pair of first-degree kinship. After employing these quality control steps, we acquired data obtained from a total of 89 Palaungic-speaking individuals with 617,132 genetic markers (Table 1).

We utilized the “bmerge” function in PLINK version 1.90b5.2 to combine our newly genotyped Palaungic-speaking data with genome-wide SNP data obtained from modern and ancient populations in East and Southeast Asia, as well as to combine reference populations like the Mbuti and French from the Allen Ancient DNA Resource (AADR) version 54.1 [16]. Additionally, we incorporated data obtained from previous studies [12, 17–22]. We then assessed the data quality using PLINK version 1.90b5.2 and excluded SNPs with more than 5% missing data, those that had less than 15,000 SNP positions, or those not in Hardy-Weinberg equilibrium with a significance level of p-value < 0.00005. This resulted in a dataset comprised of 2,390 individuals from 263 populations (Tables S2 and S2) with 499,039 SNP positions available for further analysis.

## Population structure and relationship analyses

The genetic structure and relationships of the studied sample were analyzed by pruning SNPs in the same linkage disequilibrium with an r^2^ value greater than 0.4 within windows of 200 variants and a step size of 25 variants using the “indep-pairwise” command in PLINK version 1.90b5.2. After excluding the Mbuti and French populations used as outgroups, there were 219,244 SNP positions available for analysis from a total of 2,355 individuals. Principal Component Analysis (PCA) was carried out using smartpca, a component of the EIGENSOFT package [23]. We used all default parameters along with the additional parameters of lsqproject: YES and autoshrink: YES. The ancient DNA data, which differ genetically from modern populations, were then projected.

ADMIXTURE, a model-based clustering algorithm for ancestry estimation method, was utilized to investigate the genetic composition of a merged dataset comprised of 2,390 individuals obtained from 263 modern and ancient populations. This analysis aimed to discern genetic structures and infer ancestral origins with respect to ethnicities and language groups. We employed ADMIXTURE version 1.3.0 [24], varied the number of assumed ancestral components (K) from 2 to 12, and conducted 100 bootstrap iterations with different random seeds. The optimal K value was determined by assessing the lowest cross-validation error and the highest log-likelihood using 10-fold cross-validation. Data visualization was accomplished using PONG software version 1.4.7 [25], R software [26], and AncestryPainter [27].

Furthermore, we examined drift and excess ancestry sharing within the studied populations by calculating *f*_*3*_ and *f*_*4*_-statistics using ADMIXTOOLS version 5.1 [13], in conjunction with admixr version 0.7.1 [28]. The Mbuti population was used as an outgroup for modern-modern population analysis, while the French population served as an outgroup for modern-ancient population analysis. The data were then visualized using the pheatmap package in R software version 3.6.0 to generate a heatmap. We also calculated the *F*_ST_ genetic distance between population pairs using ADMIXTOOL 2 [29], and subsequently constructed a neighbor-joining phylogenetic tree utilizing the MEGA-X software [30].

## Genetic ancestry analyses

To investigate possible ancestral origins and genetic exchanges, we initially created a TreeMix-based tree involving our focus populations and several Asian ancestral groups. The African Mbuti, European French, and Indian populations were utilized as outgroups for this analysis. To streamline the tree structure, we organized the TK populations based on their language subfamilies, whereas the ST, AN, and HM populations were grouped according to their geographic locations (East or Southeast Asia). Using the TreeMix version 1.12 program [31], we reconstructed a phylogenetic tree with migration events ranging from 0 to 3 using 10 independent runs and then selected the topology with the highest likelihood.

We proceeded to create an admixture graph, selecting backbone populations from various language families in Southeast Asia, as has been outlined in [19]. The Atayal, Dai, Cambodian, Miao, and Naxi were chosen as representative ethnic groups that speak AN, TK, AA, HM, and ST languages, respectively. We then incorporated our interest in AA-speaking populations and the specific case between the Lavue and Karen populations into the graph. For modeling the admixture graph, we utilized a dataset of 499,039 SNPs acquired from modern populations as input for ADMIXTOOLS 2 [30]. Initially, we calculated pairwise *f*_*2*_ statistics between groups using the “extract_f2()” function with specific parameters; “maxmiss = 0” (no missing SNPs for calculation), “useallsnp: NO” (allowing no missing data), and “blg = 0.05” (setting SNP block size at 0.05 morgans). Subsequently, we derived allele frequency products from the computed *f2* blocks using “f2_from_precomp()”.

Next, we explored the best-fitting admixture graph by running ten independent iterations of “find_graphs()” for each scenario. Among the 100 independent runs, we selected the one with the lowest score and then made calculations based on residuals between expected and observed *f*-statistics given the data. To validate the selected graph, we tested it using “qpgraph()” with parameters “numstart = 100, diag = 0.0001, return_fstats = TRUE”, checking if the worst-fitting Z score absolute value was below 3. Starting with no migrations (numadmix = 0), we incrementally added migrations until we reached a fitting graph, which we considered the best-fitting graph for that specific scenario.

## Results

### Population structure

**Fig. 2:**
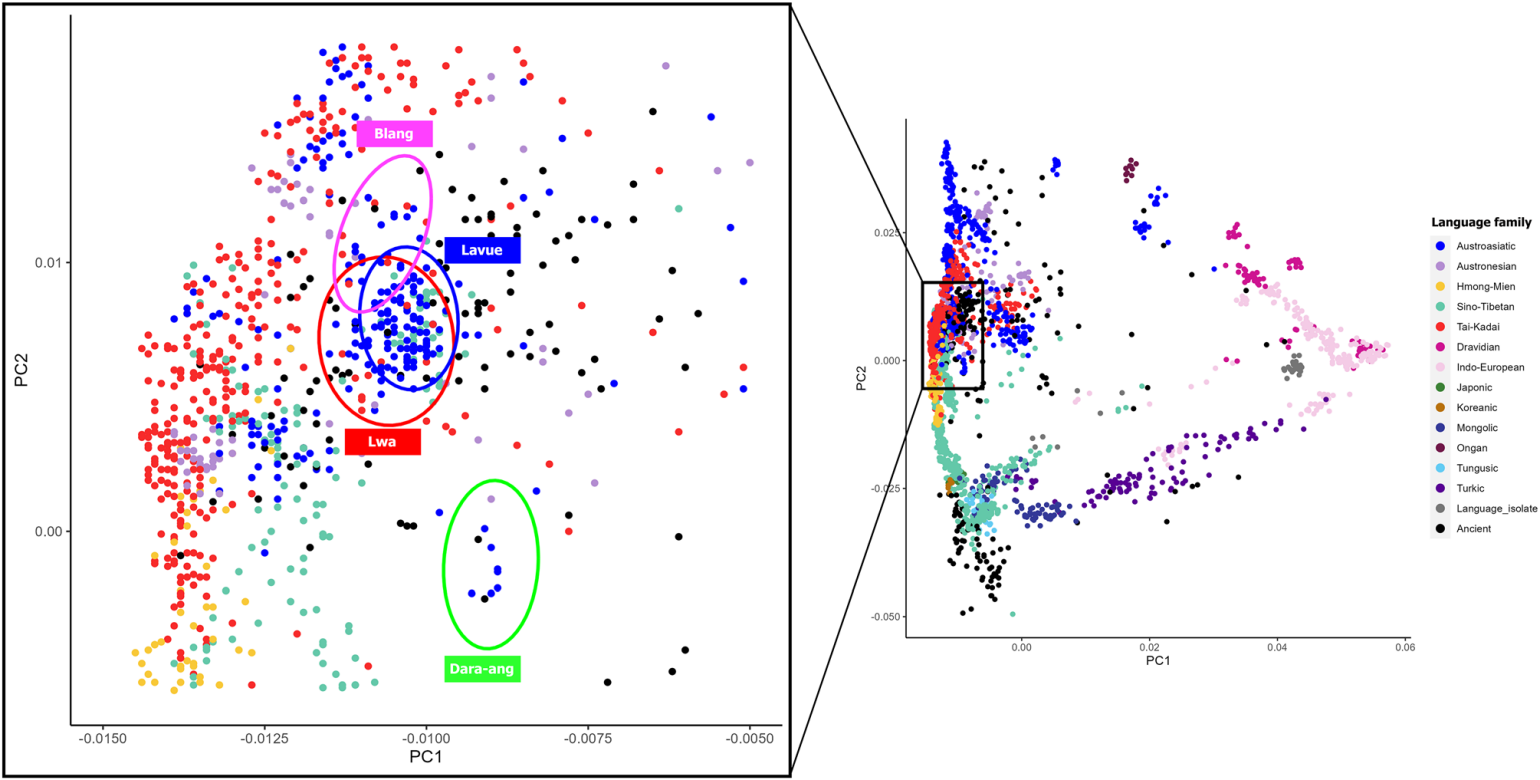
PCA plot for the genome-wide SNP data of individuals from East and South Asia. Each individual is colored by language family according to the key in the right panel. The plot focusing on the Palaungic-speaking populations is zoomed in on the left

We generated genome-wide SNP data from 89 Palaungic-speaking individuals belonging to the Lavue and Lwa ethnonyms who reside in northern Thailand (Table 1; Fig. 1). Using a set of 219,244 SNP positions, we conducted Principal Component Analysis (PCA) to explore the genetic structure and relationships among a total of 2,355 individuals, including our samples and various Asian populations (Fig. 2). The PCA analysis divided the populations into subgroups based on geographical regions. PC1 distinguished East Asians (on the left side) from South Asians (on the right side), while PC2 separated Southeast Asians (at the top) from Northeast Asians (at the bottom). Additionally, when considering subgroups by language families, populations speaking the same language tended to cluster together, except for the AA family, which was dispersed across the map. In our newly generated data, we observed that the Palaungic language group showed genetic proximity to the AA, TK, and ST families, with substantial overlap in their positions. Within the Palaungic branch, the Lavue, Lwa, and Blang samples were closely positioned with overlaps, while the Dara-ang people exhibited distinct positions.

We proceeded to examine the genetic components using the ADMIXTURE program version 1.3.0 [24], by dividing the gene pool into groups (K) ranging from 2 to 12, with each grouping repeated 100 times. The analysis indicated that the lowest cross-validation value occurred at K = 9, suggesting that 9 groups represent the most suitable number of ancestral components (Fig. S1).

**Fig. 3:**
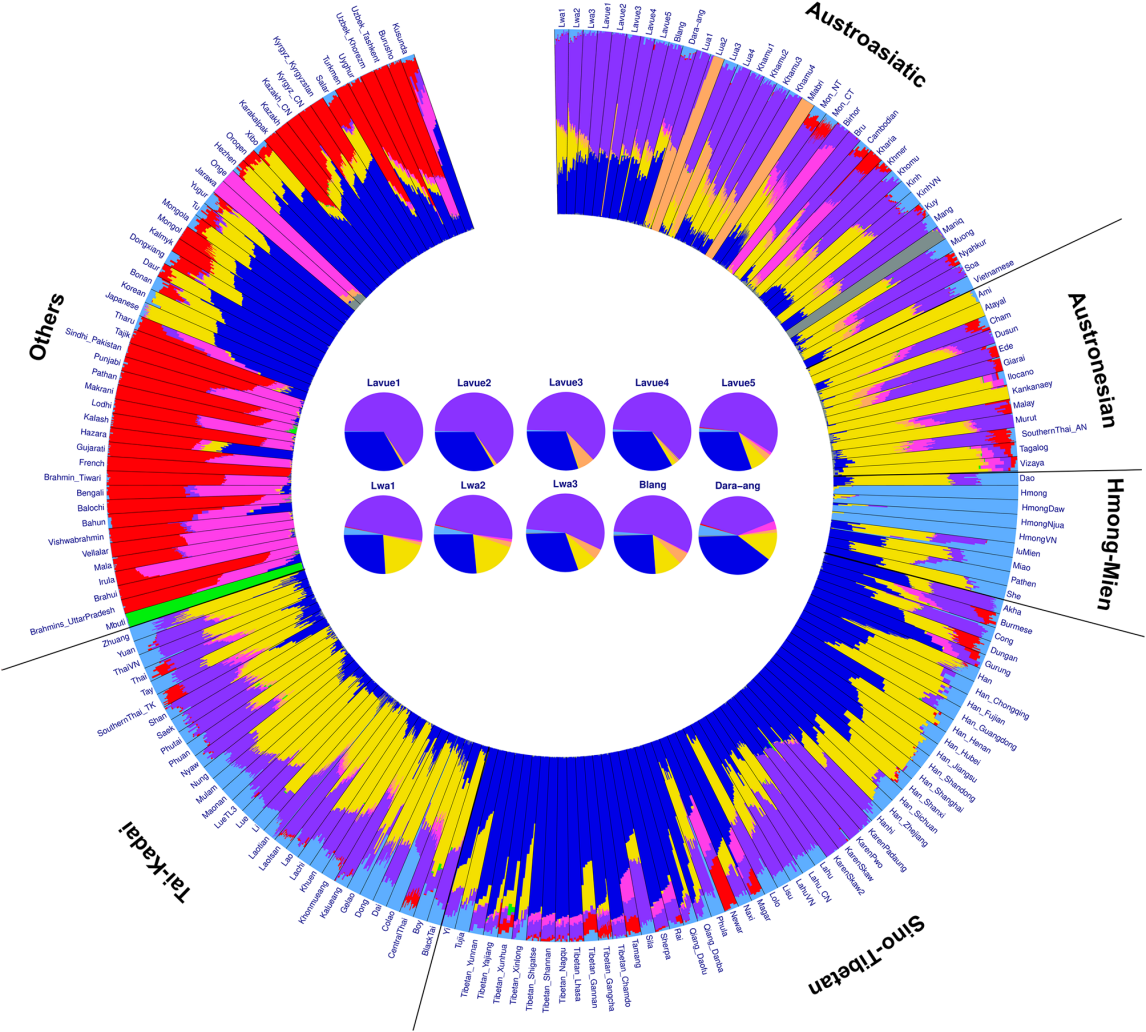
ADMIXTURE results of modern populations from East and South Asia. The plot was delineated into K = 9 groups of ancestral components. Each individual is depicted by a bar segmented into K colored sections, representing their estimated ancestry components. Populations are demarcated by black lines, and their language family is labeled outside the diagram

At K = 9 (Fig. 3), when considering language family groups, it is evident that the AA family exhibits a specific genetic component in purple, while TK and AN display a major yellow component, and HM shows the highest proportion of blue-green components. The ST family is characterized by a prominent blue component, whereas red and pink components are primarily associated with Dravidian and Indo-European language families. Moreover, some populations demonstrate unique proportions; for instance, the Mbuti people showcase a dominant green component, the Mani people have a major gray component, and the Mlabri people exhibit a major orange component.

Regarding the Palaungic branch (Fig. 3), our findings reveal that the Lwa, Blang, and Dara-ang populations exhibit three main genetic components (purple, blue, and yellow), while the Lavue population has two primary genetic components (purple and blue). These resemble the genetic components of the ST family, which includes the Karen, Lahu, and Lisu ethnic groups. Even as K increased to 12 (Fig. S2), the Lavue and those speaking ST languages continued to share similar proportions of genetic components. Additionally, upon comparison with ancient DNA, the pink component found in some populations of the AA family was observed in several ancient samples spanning from the Neolithic to the Bronze Age, albeit in varying proportions (Fig. S2).

## Genetic relationships among populations

**Fig. 4:**
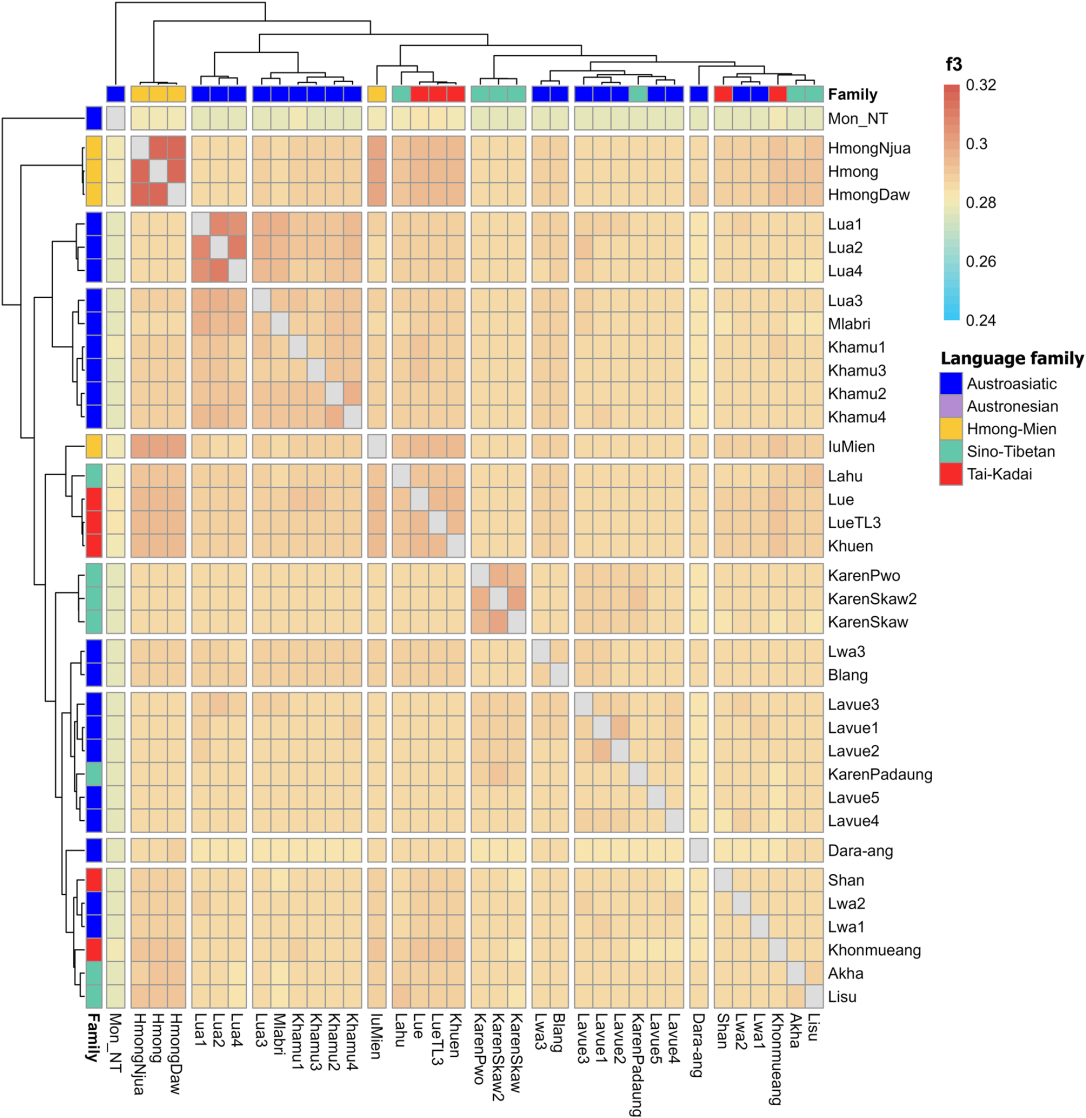
Heatmap displaying allele sharing profiles of populations in northern Thailand, as has been determined by *f*_*3*_-statistics. The colored bar located at the top-right corner indicates the statistical values and language family of each ethnic group

The analysis of genetic relationships among populations involved calculating *f*_*3*_ and *f*_*4*_-statistics. The *f*_*3*_-statistics analysis, represented as *f*_*3*_(X, Y; Mbuti), quantified the shared drift between two populations, X and Y, since their separation from an African Mbuti outgroup. A higher *f*_*3*_ value indicates a closer genetic relatedness between populations. Among the AA populations in northern Thailand, the *f*_*3*_-statistics were found to be high within the Lua ethnic group. Additionally, the Lua also showed greater allele sharing with the Khamu and Mlabri people, who belong to the Khmuic branch, as compared to the Lavue and Lwa of the Palaungic branch (Fig. 4 and Table S3). When applying the *f*_*3*_(X, Y; Mbuti) form to modern populations in Asia (represented by X) and the AA-speaking group (represented by Y), the analysis revealed close genetic relatedness within the AA language group, with some exceptions like the Maniq people and certain populations originating from India (Birhor and Kharia). Notably, the AA-speaking people in northern Thailand exhibited higher allele sharing with populations in Southern China and Southeast Asia than those from other regions (Fig. S3).

**Fig. 5:**
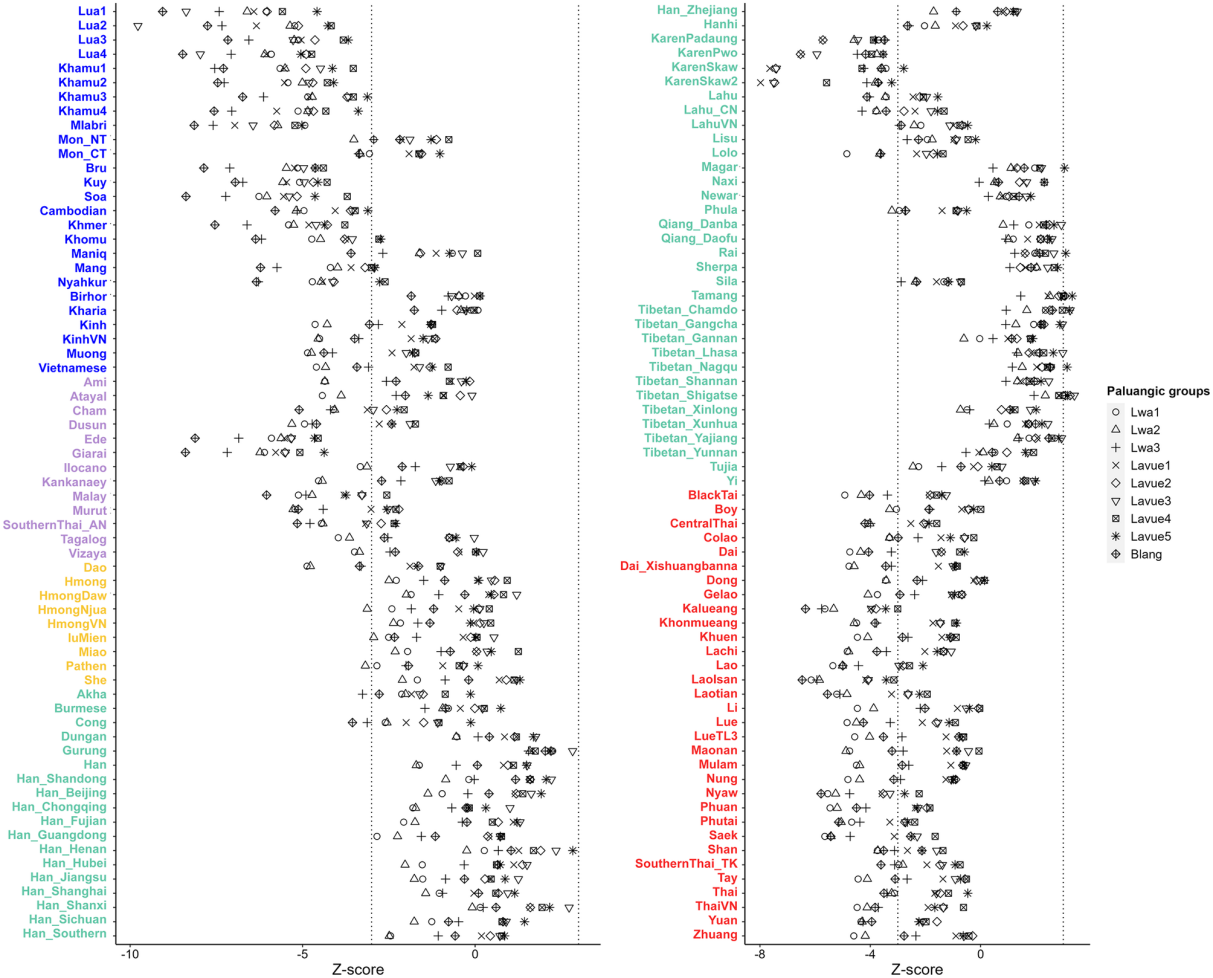
The *f*_*4*_-statistics compare Palaungic-speaking populations with ethnic groups from various language families. Z-scores are computed for *f*_*4*_(W, X; Y, Mbuti), where W represents a selected AA-speaking Dara-ang ethnic group, X represents other populations from the Palaungic branch, and Y denotes a population not in the Palaungic branch, with their ethnic names labeled on the left side. Different symbols are used to represent different populations for X. The ethnic names are labeled in colors to indicate different language families. The vertical black dashed lines denote + 3/-3

When examining population relationships using *f*_*4*_-statistics in format *f*_*4*_ (W, X; Y, Mbuti), where W represents a specific Palaungic-speaking Dara-ang ethnic group, X denotes other populations within the Palaungic branch, and Y encompasses various ethnic groups from the AA, TK, ST, AN, and HM families (Table S4), the proximity of points to the compared ethnic groups (as depicted on the left side of Fig. 5) indicates close genetic relationships. Generally, populations within the Palaungic branch demonstrated a close genetic affinity with subgroups of the AA family, particularly the Khmuic (including Khamu, Mlabri, and Lua) and Katuic (Kui, Bru, and So) branches. They also exhibited genetic relatedness with the Karenic branch of the ST family and certain AN-speaking populations (such as Ede and Giarai) in Vietnam.

In comparison to the TK family, we observed that the Lwa and Blang populations tended to have a closer relationship with TK-speaking populations than the Lavue population (Fig. 5). Additionally, when comparing the genetic data of various modern AA-speaking populations with ancient DNA obtained from Southeast Asia, all populations of the Khmuic branch showed relatedness with samples from TamPaLing in Laos and TamYappaNhae in Thailand. Interestingly, Lavue3 is the only population in the Palaungic branch that displayed significant genetic affinity with the Iron Age ancient DNA obtained from the TamYappaNhae archaeological site in Mae Hong Son Province of Thailand (Fig. S5 and Table S5).

To better understand the genetic affinity and differentiation among the studied samples, we calculated pairwise *F*_ST_ values between the target and reference populations and constructed a neighbor-joining tree (Tables S6, S7, and Fig. S6). When we grouped the studied AA populations according to their endonyms, we found that the Mlabri exhibited the greatest genetic distance from other AA-speaking populations in northern Thailand, with *F*_ST_ values ranging from approximately 0.08 to 0.10. With the exception of the Dara-ang, the Palaungic-speaking groups (Lavue, Lwa, Blang) exhibited close genetic relatedness (*F*_ST_ = 0.004–0.008). The Lua of the Khmuic branch displayed a relatively greater distant genetic relationship with the Palaungic group (*F*_ST_ ~0.01–0.02) (Table S6). In comparison with other ethnic groups in East and Southeast Asia, the Mon_NT in northern Thailand exhibited close genetic relatedness with the Mon_CT, with an *F*_ST_ value of 0.001, and was clustered with populations from other regions of Thailand such as Central Thai, Southern Thai, and Lao Isan (Table S7 and Fig. S6).

## Genetic ancestry of populations

**Fig. 6:**
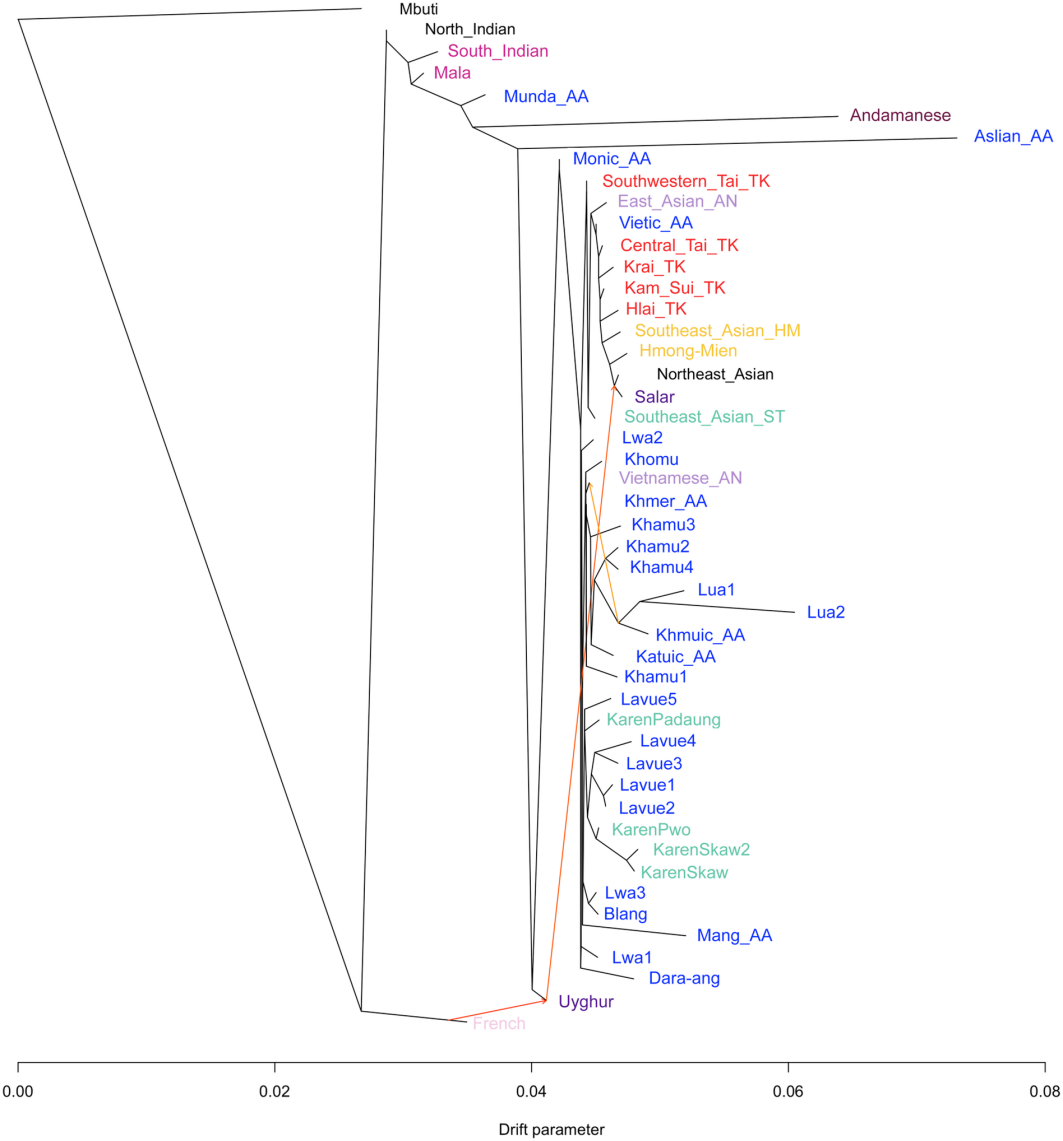
TreeMix diagram with three migration events of the AA-speaking ethnic group in northern Thailand and East Asia. Populations are labeled with different colors based on their language family

The genome-wide data of AA speakers in northern Thailand and various Asian ancestral populations, including those outside the region, were analyzed using the TreeMix version 1.12 program [31] to construct a maximum-likelihood tree. The results illustrate that the populations of the Palaungic and Khmuic branches formed a different genetic cluster (Fig. 6). Specifically, within the Palaungic branch, Lavue1-4 form a cluster along with KarenSkaw and KarenPwo, while Lavue5 clusters with KarenPadaung. The Lwa3 clusters with the Blang ethnic group, whereas the other Lwa populations exhibit diversity within the tree. When we incorporate migration events into TreeMix analyses, we observe a connection between the clade of Khmuic-speaking people in Thailand and a clade comprising some ethnic groups from Vietnam (Vietnamese and Khomu) (Fig. 6).

**Fig. 7:**
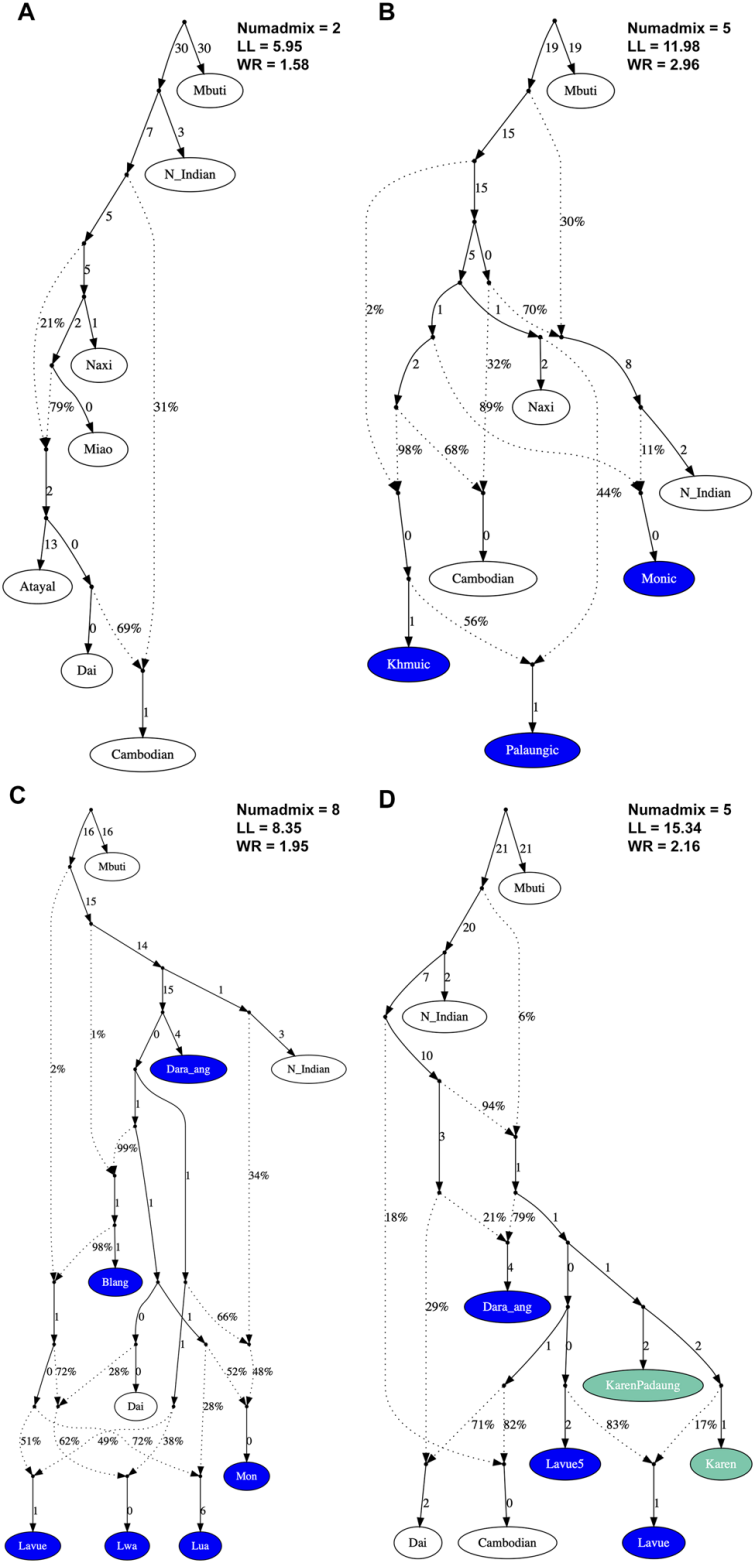
Admixture graphs of AA populations. (**A**) representatives of different language families including the Atayal, Dai, Cambodian, Miao, and Naxi for the AN, TK, AA, HM, and ST language families, respectively; (**B**) Monic-, Khmuic-, and Palaungic lingustic branch (**C**) Monic- and Palaungic-endonym; (**D**) Palaungic- and Karenic-speaking groups. White nodes denote backbone populations. Dashed arrows represent admixture edges, while solid arrows indicate drift edges reported in units of *F*_ST_ × 1,000

We delved deeper into ancestry by initially constructing backbone admixture graphs using representatives from the AA, TK, AN, HM, and ST language families, namely the Cambodian, Dai, Atayal, Miao, and Naxi, respectively, with the Mbuti serving as the outgroup. We then incorporated AA speakers from northern Thailand into the analysis and identified the best-fitting graph (Fig. 7C). Upon isolating the outgroup, the AA-related ancestry was separated from the North Indian group and emerged as the Dara-ang, followed by the Blang. The Lwa presented as admixed populations that incorporated AA-related and Dai-related ancestries in proportions of 38% and 62%, respectively. Subsequently, we delved further into the point of interest to determine the ancestry of the Lavue and Karen populations, which indicated genetic closeness. We incorporated Lavue, Karen, and Dara-ang into the graph, grouping them based on TreeMix clustering: Lavue (Lavue1-4), Lavue5, KarenPaduang, Karen (KarenSkaw and Karen Pwo), and Dara-ang. We observed shared ancestry between the Lavue and Karen populations. This ancestral lineage then diverged to form the Dara-ang group, which exhibited a greater influence from North Indian-related ancestry. The Lavue populations emerged as a result of a mixture between Lavue-related and Karen-related ancestries, with a ratio of 83% and 17%, respectively (Fig. 7D).

## Discussion

In the past, many AA-speaking individuals in northern Thailand felt embarrassed about their identity and often tried to conceal it by assimilating into the majority Northern Thai population [10]. This situation has created confusion for historians and scientists studying these communities. However, with the growing acceptance of ethnic diversity in the modern world, this trend is changing. While these individuals consider themselves Thai, they also take pride in their own unique culture and language. This changing dynamic presents an opportunity to clarify and correct the AA ethnic identity, and we encourage the scientific community to use their self-identified ethnonyms, thereby acknowledging their valuable contributions to the field of genetic anthropology.

The populations of AA-speaking language in northern Thailand are linguistically linked with a structure within the Mon-Khmer subfamily [7], suggesting a shared ancestry that has persisted over time, evidenced by their close relationships and the similar genetic components we observed in our study (Fig. 2). The primary genetic component identified in AA speakers of northern Thailand (represented as the purple component in Fig. 2) is likely linked to Neolithic agriculturalists in Southeast Asia. This component is also prevalent in ancient Neolithic DNA obtained from the region, such as N_GuaChaCave, N_ManBac, and N.BA_TamPaLing (Fig. S2). Previous studies have shown a genetic connection between Neolithic rice farmers in southern China, particularly in the Yangtze and Yellow River Basins, and the spread of Southeast Asian agriculturists, who are considered the ancestors of modern AA-speaking people [32]. This link is supported by the presence of specific Y-chromosomal haplogroups, notably O1 and its related sublineages [33], indicating the significant influence of ancient rice farmers on the gene pools of populations in Southern China and Southeast Asia. The O1b1a1a-M95 haplogroup, predominant among AA speakers in Thailand and Laos with a frequency exceeding 70% [34], has been detected in approximately 3,000-year-old individuals from the Wucheng site in Jiangsu along the Yangtze River Basin, from the Hengbei site in Shanxi, and in several 1,500-year-old individuals from Guangxi. Between 3,000 and 4,500 years ago, proto-AA-speaking ancestors migrated from southern China to northern Vietnam, carrying shouldered stone tools and domesticated rice. This migration eventually extended into Mainland Southeast Asia and Northeast India, leading to the formation of various ethnic groups, including AA speakers in northern Thailand [35].

However, due to the fact that AA speakers in Thailand are fragmented and have largely assimilated into mainstream Thai society, our results do not show a direct genetic link to the earliest rice farmers in southern China (Figs. S4 and S5). Extreme fragmentation and the significant loss of AA ancestry lineages have also resulted in the appearance of unidentified intermediate nodes in the admixture graph analysis (Fig. 7). Our findings suggest complex ancestries for AA populations in Thailand; however, further research, including broader sampling of modern populations and ancient DNA, would be needed to clarify their ancestry.

Despite the retention of Neolithic agriculturalist ancestry among most AA speakers in northern Thailand, genetic diversity within these groups indicates significant gene flow from neighboring populations. The spread of agricultural practices facilitated the movement of AA populations and associated genetic admixture across regions. This process has contributed to the genetic substructure observed among AA speakers in northern Thailand, which will be further discussed with regard to their specific language branches.

### Monic branch

The Mon people are historically recognized as one of the earliest settlers in Mainland Southeast Asia, with their civilization centered in Central Thailand and Southern Myanmar dating back over a thousand years. They are renowned for their sophisticated art, architecture, Buddhism, and literary works, which have significantly influenced the cultural development of the Thai and Burmese people. The Mon established the renowned Dvaravati Kingdom, spanning from Southern Burma (Myanmar) to Central Thailand between the 3rd and 10th centuries AD. By the 8th century AD, their civilization extended to the Haripunchaya Region in present-day Lamphun Province, Northern Thailand [8]. However, due to displacement and assimilation by the TK-speaking groups over centuries, only a small number of individuals still identify as Mon in contemporary times.

Although the Mon and the local inhabitants referred to as “Lua” have been consistently mentioned together throughout the history of northern Thailand [36, 37], they have generally been recognized as distinctly different ethnic groups. The genetic distinction of the Mon in northern Thailand from other AA-speaking groups appears to have been influenced by South Asian admixture, as indicated by the pink and red components in the ADMIXTURE analysis (Fig. 2) and the North Indian-related ancestry observed in the admixture graph (Fig. 7). Previous studies have shown that both AA- and TK-speaking populations in Southeast Asia exhibit significant genetic contributions from South Asian populations, suggesting ancient gene flow between these regions. This was likely facilitated by trade routes and the spread of Buddhism from the Indian subcontinent [20]. The close genetic relationship between the Mon population in northern Thailand and the Mon in the central region, as evidenced by the *F*_ST_ genetic distance (Table S7 and Fig. S6), aligns with their shared Southern Mon-Khmer language, cultural customs, and Buddhist practices.

### Khmuic branch

The AA-speaking population in northern Thailand, belonging to the northern Mon-Khmer subfamily, can be broadly categorized into two primary branches: the Khmuic branch, concentrated mainly along the Thailand-Laos border with a notable presence in Nan Province, and the Palaungic branch, primarily situated in Chiang Mai and Mae Hong Son Provinces in the western part of the region (Fig. 1). Some ethnicities from these two branches are collectively referred to as the “Lua.“, and it remains an open question as to whether they have any kin relations [38]. However, these groups differ significantly in terms of language and the realization that they belong to distinct branches of the AA family, as well as in their cultural practices and post-marital residence patterns. Traditionally, Lavue communities in Chiang Mai and Mae Hong Son follow a patrilocal residence system, where the bride moves to live with the husband’s family after marriage, while Lua communities in Nan practice a matrilocal residence system, tracing their lineage through the female lineage [8].

Although the Lua, Lwa, and Lavue groups share the main genetic composition of the AA family, the Lua people in Nan Province exhibit a relatively greater distant genetic relationship from the others and seem to share a genetic ancestry with various local Khmuic-speaking populations such as the Htin, Khamu, and Mlabri (Fig. 6). Our study also supports the genetic affinity between Khmuic-speaking communities and the Katuic, another AA branch found in northeastern Thailand [19]. Comparisons with other modern populations and ancient DNA in Southeast Asia show relatedness between the people of the Khmuic branch and AA-speaking Mang and Khomu ethnicities in Vietnam (Figs. S4 and S6), as well as connections to ancient DNA obtained from the TamPaLing Bronze-age site in northern Laos and the YapPaNhae Iron-age site in northern Thailand (Fig. S5). While direct historical connections are unclear, the distribution of Khmuic-related ancestry across modern and ancient populations in Thailand, Laos, and Vietnam supports the hypothesis that they share ancient ancestry [12, 39, 40]. The evidence of a genetic connection observed between the Khmuic population residing in the eastern part of Northern Thailand and the ethnic communities found in Laos and Vietnam has been absent in the Palaungic-speaking groups in the western part of the region. This difference could contribute to the genetic differentiation between the people of the Khmuic and Palaungic branches.

In line with previous findings [12], we can confirm that the Lua people in Nan Province are the same ethnic group previously identified as the Htin in earlier genetic studies [2, 41–44]. The confusion created by assuming that the Lua people belong to the Htin ethnicity stems from differing perspectives. Outsiders, government officers, or other lowland communities typically refer to the indigenous people in this area as Htin (meaning “local people” in literal terms). However, the Lua people identify themselves as Lua, and many of them find being called Htin derogatory. Although the term Lua is preferred out of respect for the community, the term Htin is still officially recognized as a hill tribe by the Thai government [45]. It is also important to note that some works use alternative names for the Lua (Htin) in Nan Province, such as Mal and Pray (Prai), which linguistically denote two subgroups of the Lua. While they are distinguished by their ceremonial practices, and it has been suggested that they were separated lineage-wise a few hundred years ago [46], there is currently no genetic evidence available to differentiate between these two Lua subgroups.

### Palaungic branch

The majority of Palaungic speakers in northern Thailand are concentrated in Chiang Rai, Chiang Mai, and Mae Hong Son Provinces, as they reside in the mountainous areas near the Myanmar border. Our analysis using PCA and *f*_*3*_-statistics (Figs. 2 and 4) revealed close genetic affinities among most Palaungic-speaking communities, except for the Dara-ang people. An examination of the genetic components has revealed that all studied Palaungic-speaking populations have significant genetic contributions from two main sources: the purple component, commonly found in AA-speaking groups, and the blue component, prevalent in ST-speaking groups, particularly the Tibeto-Burman subfamily (Fig. 3 and S2).

Tibeto-Burman-speaking people exhibit high ethnolinguistic diversity and are widely dispersed across regions from the Tibetan Plateau to lowland South China and Southeast Asia. Recently accumulated whole-genome data have revealed a strong genetic link between the Tibetan-Yi corridor and ancient Yellow River populations, supporting a Northern China origin for the Tibeto-Burman people [47]. These groups also share genetic ancestry and are influenced by varying levels of admixture with the Han Chinese and northern minority groups in East Asia [48]. Over millennia, Tibeto-Burman populations migrated southward to southern/southwestern China, where they intermingled with various populations, leading to significant historical interactions and admixture [49]. Eventually, they settled in the mountainous regions of northern Thailand and Myanmar. Coexistence between Palaungic and Tibeto-Burman speakers, such as the Akha, Lahu, and Lisu in the western part of northern Thailand, may explain the higher proportion of ST-related ancestry within the Palaungic branch when compared with the Khmuic branch, contributing to genetic differentiations between these groups (Fig. 3).

Although all populations belonging to the Palaungic branch consist of two main ancestries, AA-related and ST-related, their within-group substructure that is driven by different ancestry compositions is observed as follows.

### Dara-ang

The Dara-ang is the endonym of the people who are usually known as Palaung, a Burmese language exonym. In China, they are known as De’ang and live in the southwestern part of Yunnan Province. The Dara-ang are believed to be the first settlers on the upper bank of the Salween River before the arrival of any other Palaungic-speaking people [8]. There are now more than 250,000 people in Myanmar, but there are only about 1,937 persons living in Thailand. These people are mostly immigrants who had migrated in 1983 AD and settled in Chiang Mai Province next to the country’s border. Although there are many subgroups of the Dara-ang, such as Pale (or Silver), Shwe (or Golden), Rumai, and Riang, all those residing in Thailand belong to the Pale Dara-ang [8].

Our genome-wide analysis has revealed that the Dara-ang group stands out as the most distinct among the Palaungic relatives (Figs. 2 and 4). The ADMIXTURE diagram reveals that the Dara-ang possess unique pink genetic elements, a feature uncommon in other Palaungic-speaking ethnicities (Fig. 3). When compared with other Asian populations, this pink genetic component is similar to that which was found in the 100BP_GreatAndaman ancient DNA and among indigenous hunter-gatherer communities such as the Onge, Jarawa, and Birhor tribes in the Andaman Islands and the Indian subcontinent (Fig. 3 and S2). Previous research [35] has reported a genetic link between AA speakers and early Southeast Asian hunter-gatherers who are genetically similar to Andamanese populations. The presence of Andamanese/Indian ancestry in the Dara-ang gene pool may contribute to their distinctiveness from other Palaungic relatives. However, our modern and ancient DNA comparisons using *f*_*4*_-statistics (Fig. S5) did not confirm a close link between the Dara-ang and ancient DNA sources across Southeast Asia. This could be due to the small proportion of pink colored ancestry in the Dara-ang or the limited availability of ancient DNA data obtained from this region. Expanding the comparison to include more ancient DNA data from Southeast Asia could help clarify the origins of this genetic lineage observed in the Dara-ang population and its connection to ancient ancestry from the Indian subcontinent.

### Lavue

Since the Lavue and Lwa lack a written language, there are no documented records detailing their settlements and migrations. However, oral traditions recount a narrative suggesting that the Lavue and Lwa were originally part of the same ethnic group that once inhabited a city located in the Ping River Plain, situated in the modern-day Chiang Mai-Lamphun Basin of northern Thailand. They then were overcome by the incoming TK-speaking migrants, which resulted in the Lavue migrating to the mountains while the Lwa remained under TK governance. Historical documents from later periods refer to these indigenous peoples by various names such as Lua, Lawa, Milakhu, Tamilla, and La [37].

In our research, we have primarily focused on using the self-identification endonyms to categorize the Palaungic branch. Among those previously identified as part of the “Lua” ethnicity, we discovered two major sub-groups: the Lavue residing in mountainous areas and the Lwa residing in lowland river plains. Through the analysis of genome-wide autosomal markers, we have observed differences in ancestry composition between these two sub-groups. The Lavue maintained a genetic structure characteristic of the AA family (represented by purple and blue colors), with minimal genetic admixture with other ethnic groups (Fig. 3 and S2). While linguistic evidence has indicated that dialectical variations exist within the Lavue’s language (described as the Western Lawa in [10]) with up to eight dialects, our genetic findings reveal shared ancestry and similar genetic components within this group.

We observed an intriguing genetic affinity between the Lavue and the Karen ethnic group of the ST family (Fig. 6). Despite cultural and linguistic differences between the Lavue and Karen communities, they have developed close proximity over time. Over the past 150 years, Sgaw Karen individuals from Myanmar have migrated into the Lavue mountains in Thailand, coexisting with members of the Lavue population [10]. While intermarriage with non-Lavue individuals has traditionally been rare due to the Lavue community’s adherence to their habitat and culture, such limitations have gradually diminished in contemporary times. Evidence of intermarriages between Lavue individuals and spouses from different ethnic groups, such as Sgaw Karen and Northern Thai, supports this trend [9].

### Lwa

The Lwa group represents the Palaungic-speaking indigenous population that reside in the lowland river plains of northern Thailand. Linguistically, most Lwa individuals are clustered with the Eastern Lawa and have been noted to understand the Lavue’s language at only about 50% comprehension [10]. Our results have also revealed that the genetic structure of the Lwa group in Chiang Mai Province differs from that of the Lavue group (Fig. 4). This difference is attributed to genetic admixture, as has been evidenced by the presence of the genetic composition present in the TK family, which is rare in the Lavue group (Fig. 3 and S2). The historical relationship between the Lwa people and TK-speaking groups has been well-documented in various historical documents, which describe the Lwa as the original inhabitants of the Chiang Mai-Lamphun Basin before the incoming migration of the TK people around the 13th century [6]. The Lwa who still exist in the river plains have not maintained their original AA culture as they have been acculturated and assimilated by the TK community living around them [8]. While some Lwa communities in different districts near Chiang Mai City still acknowledge themselves as the Lwa, they have largely adopted the culture of the TK community and may eventually lose their identity in the future.

It seems that the differences in ancestry compositions between the Lavue and Lwa correspond well with their classification based on spoken language, as the Western Lawa [ISO code: lcp] and Eastern Lawa [ISO code: lwl] languages, respectively [7]. However, there is an exception with the Lavue5 from Bo Luang Village, Chiang Mai Province, which does not fit this categorization. The residents of this village identify themselves with the Lavue endonym, similar to those residing in the mountainous areas, but they are linguistically classified as Eastern Lawa alongside the Lwa in the lowland river plain. Our analysis also indicates that the genetic structure of Lavue5 resembles that of the Lwa, showing a high degree of admixture with the TK gene pool. This suggests that, within the Palaungic branch, self-identification endonyms and linguistic classifications do not solely reflect genetic relatedness.

Interestingly, we have compared our genetic results with those of animism practices and have identified a correlation between genetic clustering patterns of Palaungic speakers and differences in spiritual worship practices within their communities. Lavue populations with minimal or no evidence of TK admixture (Lavue1-4) (Fig. 3) primarily engage in the spirit ceremony centered around the “Sao Sa Kang” (the village’s sacred pillar), a landmark of animism. On the other hand, populations showing evidence of TK admixture (Lavue5, Lwa1-3, Blang) lack sacred pillars but engage in rituals honoring the spirit shrine and Buddhism [50]. This suggests that the introduction of Buddhism through contact with TK-speaking people has led to a decrease in spiritual practices among the latter group. Additionally, the degree of contact and admixture with TK people correlates with geographic locations, with populations found in lowland river plains exhibiting a higher degree of TK admixture when compared with those residing in mountainous areas (Figs. 1 and 3).

### Blang

The Blang are another ethnic group that uses the Palaungic language and is sometimes classified as being of “Lua” ethnicity. Their original homeland was in the area along the border between Myanmar and China. They are also known by other ethnonyms such as Bulang, Sam Tao, and Tai Loi. While there is no clear evidence of the Blang people’s migration to Thailand, it is estimated that they began moving there less than 100 years ago [8]. Over the past 20–30 years, the Blang people have been identified as part of the “Lua” ethnic group by Thai government officers, being recognized as one of the 13 expanded official hill-tribes of Thailand, which include the Akha, Dara-ang, Hmong, Htin, Iu-Mien, Kachin, Karen, Khamu, Lahu, Lisu, Lua, Mlabri, and Shan [45].

Using genome-wide data, we have found that the genetic structure of the Blang people is similar to that of the Lwa group, mainly comprising genetic components similar to the others of the AA family but with admixture from the TK gene pool (Fig. 3). The Blang people are an ethnic group that has had close historical interactions with other ethnic groups, particularly the TK-speaking Shan (Tai Yai) ethnicity, mainly through the trade of various daily life items, which has likely led to assimilation between these groups.

## Conclusion

We have utilized genome-wide data to analyze the genetic clustering patterns of AA-speaking ethnic groups in northern Thailand. Our findings are consistent with existing linguistic classifications, highlighting different genetic compositions among the three branches of the Mon-Khmer subfamily within the AA family: Monic, Khmuic, and Palaungic. Although the term “Lua” has been ambiguously used to refer to groups from both the Khmuic and Palaungic branches, our genomic data clearly indicate that Khmuic-speaking Lua communities are relatively genetically distant from the Palaungic-speaking Lavue and Lwa groups.

Within the Palaungic branch, the Dara-ang stand out as the most genetically distinct, retaining some remnants of ancient genetic composition. Lavue populations, mainly residing in mountainous areas, display a genetic makeup unique to the AA family and share genetic closeness with the Karenic of the ST family. On the other hand, the Lwa and Blang, residing in lowland river valleys, show genetic components resulting from admixture with TK-speaking ethnic groups. While self-identification ethnonyms and linguistic classifications can roughly categorize Palaungic-speaking populations in northern Thailand, we propose that genetic relationships are more closely linked to spiritual practices (the presence or absence of sacred pillars in communities) and geographic locations (mountainous or lowland areas).

## Electronic supplementary material

Below is the link to the electronic supplementary material.

Supplementary Material 1

Supplementary Material 2

## Data Availability

The datasets generated and analyzed during the current study are available in the Zenodo repository (10.5281/zenodo.10957983). Data can be made available upon electronic request to the corresponding authors after confirming that the data will only be used in accordance with the restrictions of informed consent, including the following: the data will not be transferred to anyone else; the data will only be used for genetic/anthropological studies but not for health or disease-related studies or for any commercial purpose; and no attempt will be made to identify any of the sample donors.
