# Supplementary material for: Under the name of “Lua”: revisiting genetic heterogeneity and population ancestry of Austroasiatic speakers in northern Thailand through genomic analysis
Source: BMC Genomics. 2024 Oct 14;25:956. doi: 10.1186/s12864-024-10865-3 (PMC11472482; doi:10.1186/s12864-024-10865-3)
Supplement: Supplementary file 2 — Supplementary Material 2 [file 12864_2024_10865_MOESM2_ESM.docx]

**Supplementary information**

**Fig. S1 Cross-validation values of ADMIXTURE ranging from K=2 to 12.**

**Fig. S2 ADMIXTURE diagram showing the genetic components of various East and South Asian populations.** K values ranging from 2 to 10 divided into groups from K=2 to K=12 for modern populations (left) and ancient DNA (right). Each individual is represented by a bar divided into K colored segments, indicating their estimated membership fractions in each of the K ancestry components. Populations are separated by black lines and their names are labeled in colors showing different language families.

**Fig. S3 Heatmap showing allele sharing profiles based on *f_3_* statistics comparing the AA speaking populations with other Asian ethnic groups**. The colored bar on the top-right indicates the statistical values, while that on the low-right indicates the language family of each ethnic group.

**Fig.** **S4 Heatmap showing allele sharing profiles based on *f_3_* statistics comparing the AA speaking populations with ancient DNA.** The colored bar on the right indicates the relevant statistical values.

**Fig. S5 Diagram illustrating the relationship between populations analyzed using *f_4_*-statistics, thereby comparing modern populations with ancient DNA in Southeast Asia.** Accordingly, *f_4_* statistics compare the AA speaking populations labeled on the left to the ancient samples from Southeast Asia on the top grey bar. Z-scores are for *f_4_* (ancient sample, Han Chinese; ethnic population, French). The vertical grey lines denote 0. Empty circles denote nonsignificant Z-scores (|Z|=<3) and solid circles denote significant Z-scores (|Z|>3)

**Fig. S6 Neighbor-joining tree of target and reference populations constructed based on the pairwise *F*_ST_ genetic distance.** The colored circles indicate the language family of each population according to the key on the top-right.

**Fig. S7 TreeMix diagram for the AA-speaking ethnic group in northern Thailand and other modern populations in East and Southeast Asia.** (A) without migration event (B) one migration event (C) two migration event (D) three migration event. Populations are labeled with different colors based on their language family.

**Table S1 General information concerning populations and samples studied.**

**Table S2 Summarized data of populations used for comparison.**

**Table S3 Results of the *f_3_* (X, Y: Outgroup), where Outgroup = Mbuti**

**Table S4 Results of the *f_4_* (Dara-ang, Palaungic-speaking group: Asian, Outgroup), where Outgroup = Mbuti**

**Table S5 Results of the *f_4_* (ancient samples, Han Chinese: ethnic groups, Outgroup) with transversion only.**

**Table S6 Pairwise *F_ST_* values between populations belonging to the Austroasiatic family in northern Thailand.**

**Table S7 Pairwise *F_ST_* values between target population and other reference populations.**


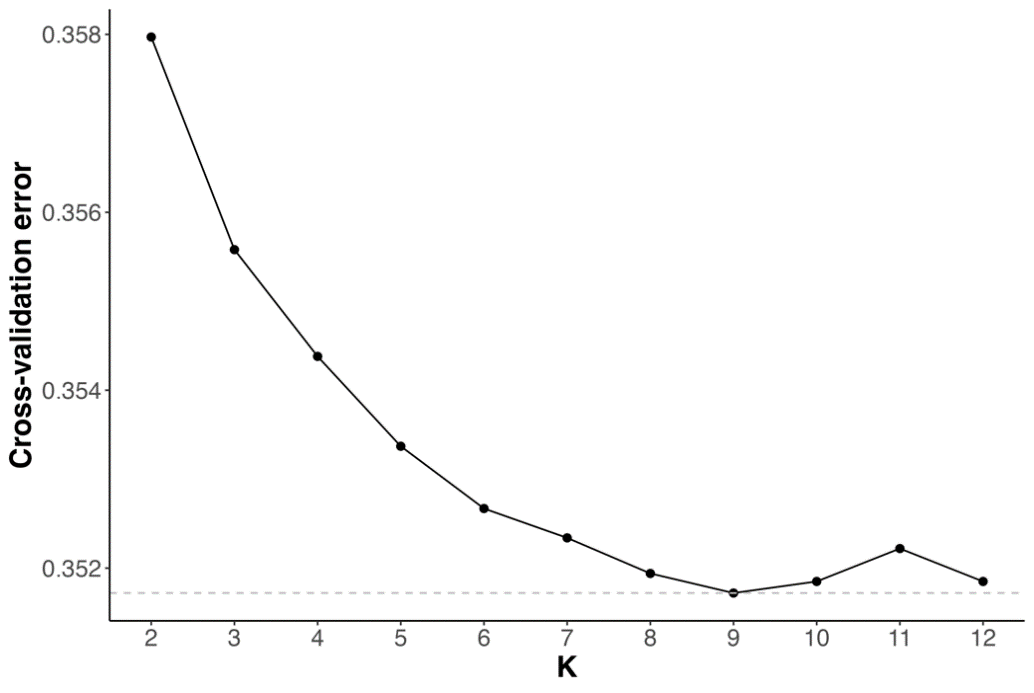


**Fig. S1 Cross-validation values of ADMIXTURE ranging from K=2 to 12.**


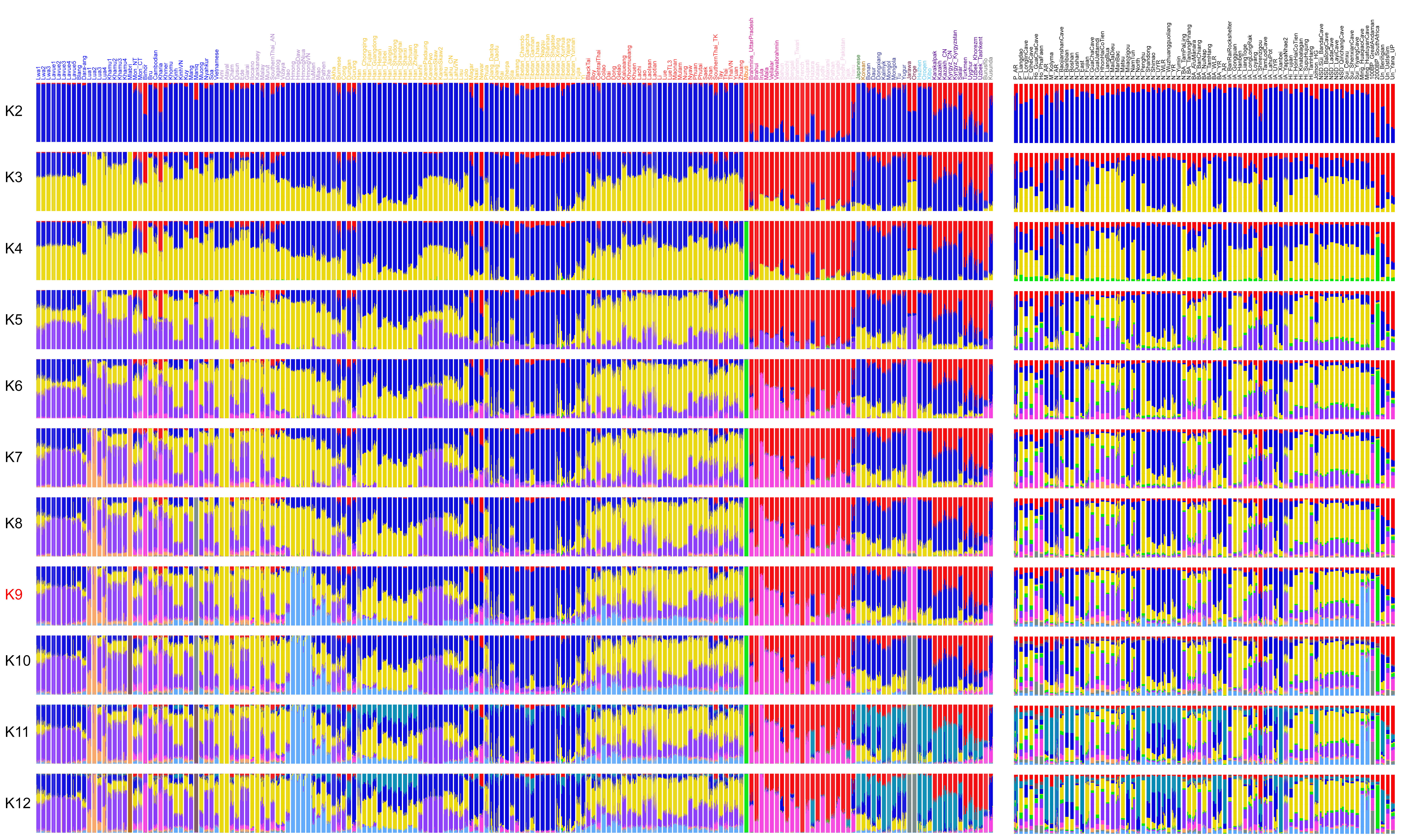


**Fig. S2 ADMIXTURE diagram showing the genetic components of various East and South Asian populations.** K values ranging from 2 to 10 divided into groups from K=2 to K=12 for modern populations (left) and ancient DNA (right). Each individual is represented by a bar divided into K colored segments, indicating their estimated membership fractions in each of the K ancestry components. Populations are separated by black lines and their names are labeled in colors showing different language families.

**

**

**Fig. S3 Heatmap showing allele sharing profiles based on *f_3_* statistics comparing the AA speaking populations with other Asian ethnic groups**. The colored bar on the top-right indicates the statistical values, while that on the low-right indicates the language family of each ethnic group.


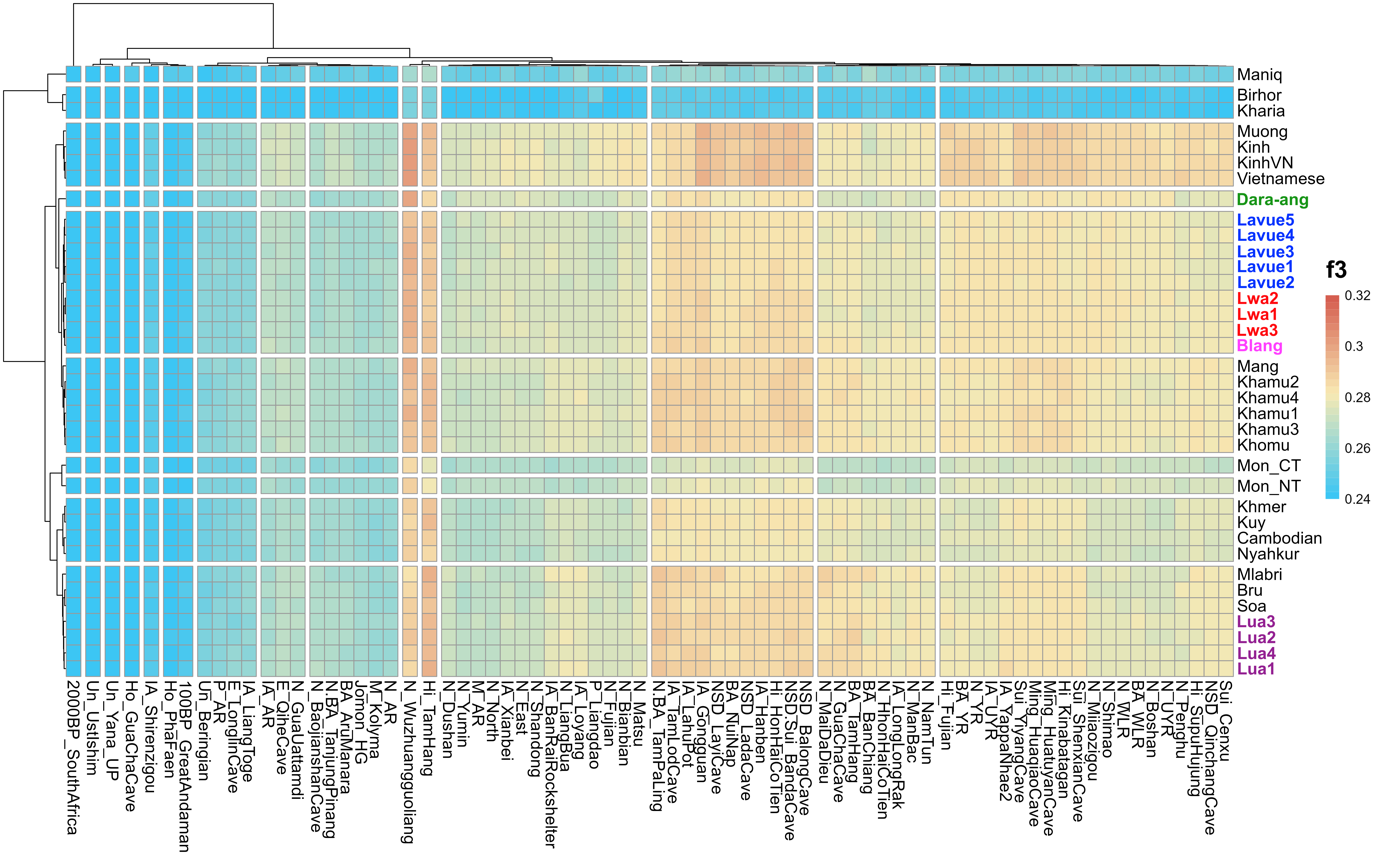


**Fig.** **S4 Heatmap showing allele sharing profiles based on *f_3_* statistics comparing the AA speaking populations with ancient DNA.** The colored bar on the right indicates the relevant statistical values.


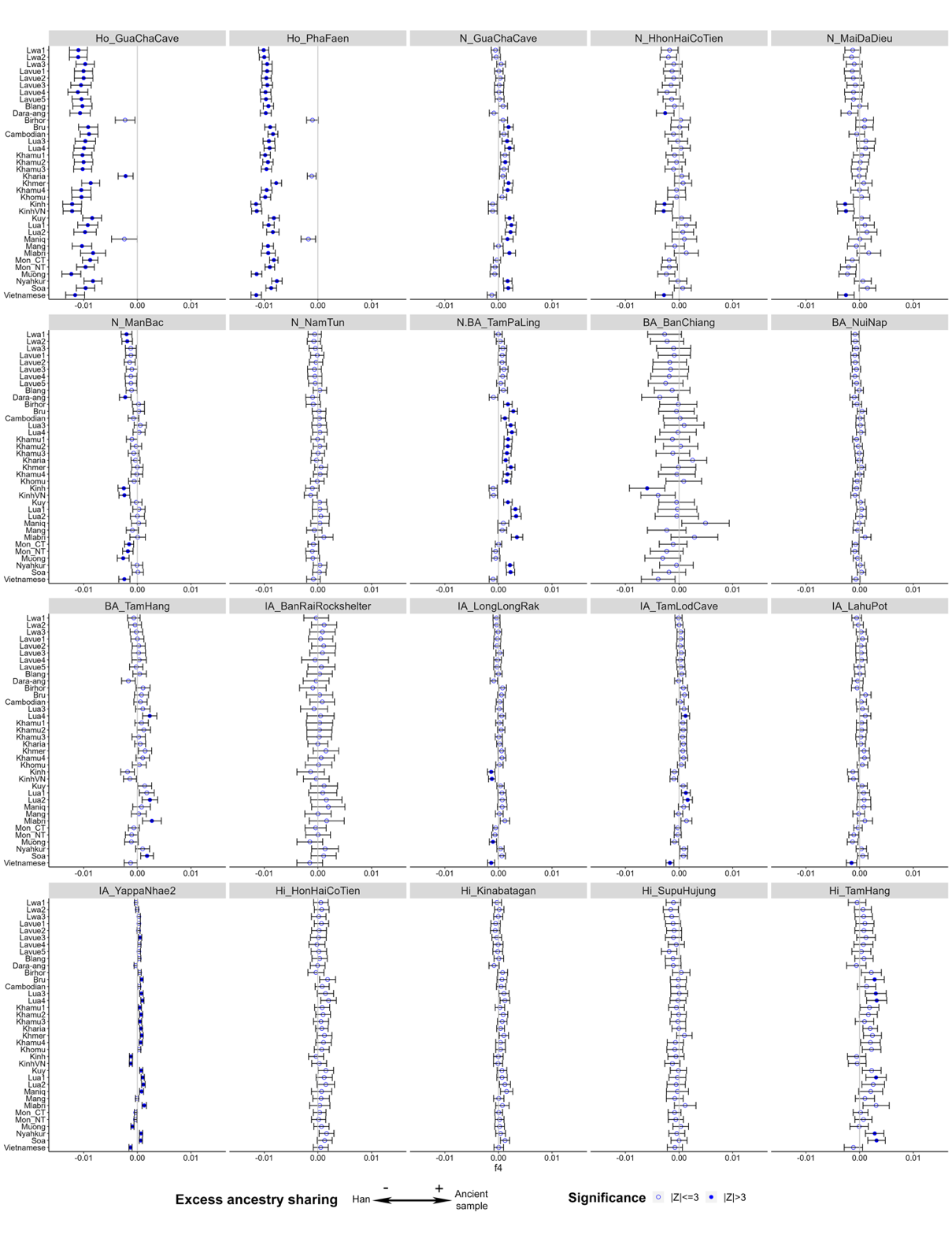


**Fig. S5 Diagram illustrating the relationship between populations analyzed using *f_4_*-statistics, thereby comparing modern populations with ancient DNA in Southeast Asia.** Accordingly, *f_4_* statistics compare the AA speaking populations labeled on the left to the ancient samples from Southeast Asia on the top grey bar. Z-scores are for *f_4_* (ancient sample, Han Chinese; ethnic population, French). The vertical grey lines denote 0. Empty circles denote nonsignificant Z-scores (|Z|=<3) and solid circles denote significant Z-scores (|Z|>3)

**
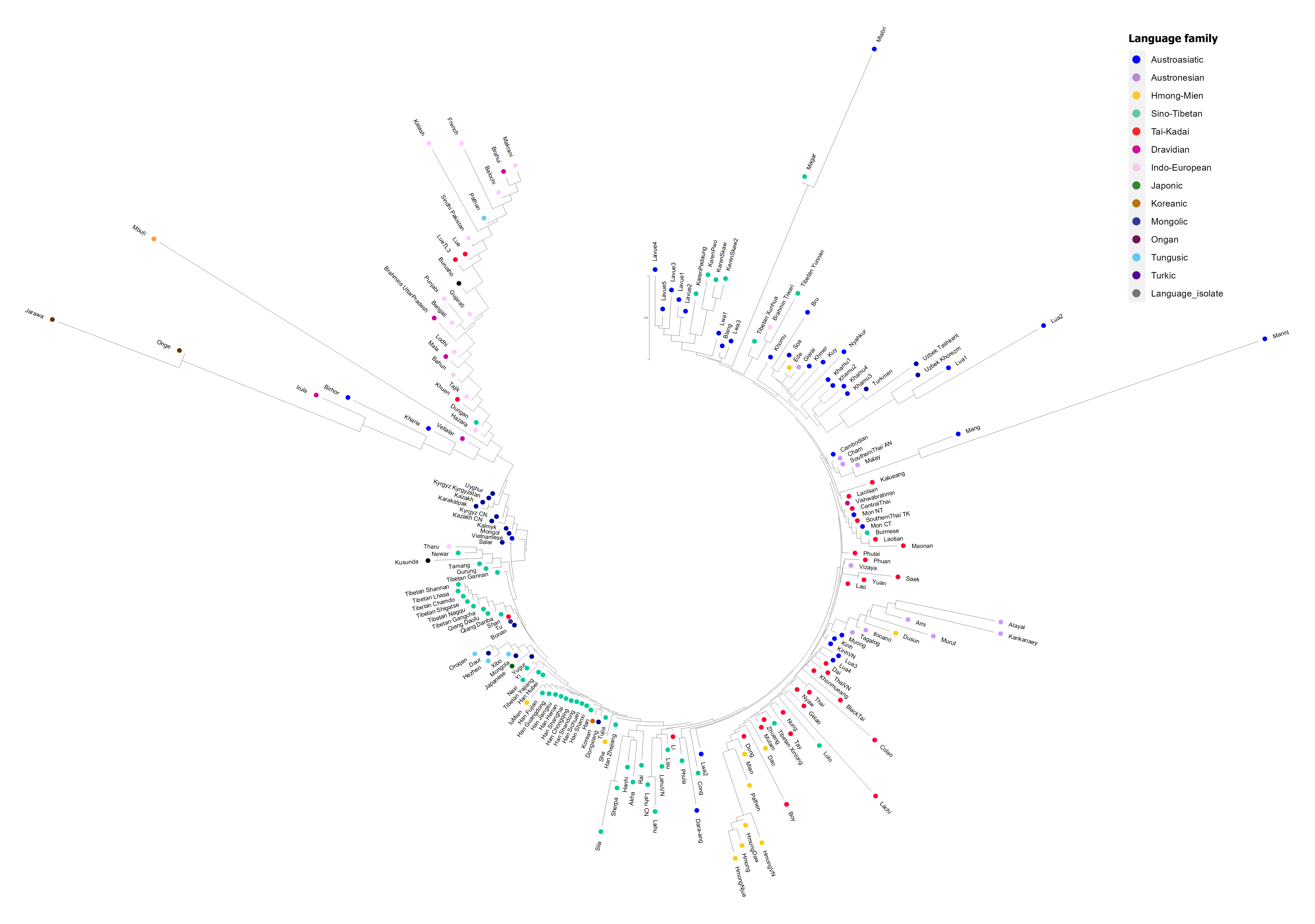
**

**Fig. S6 Neighbor-joining tree of target and reference populations constructed based on the pairwise *F*_ST_ genetic distance.** The colored circles indicate the language family of each population according to the key on the top-right.





**Fig. S7 TreeMix diagram for the AA-speaking ethnic group in northern Thailand and other modern populations in East and Southeast Asia.** (A) without migration event (B) one migration event (C) two migration event (D) three migration event. Populations are labeled with different colors based on their language family.
